# Radiation Therapy in the German Hodgkin Study Group HD 16 and HD 17 Trials: Quality Assurance and Dosimetric Analysis for Hodgkin Lymphoma in the Modern Era

**DOI:** 10.1016/j.adro.2022.101169

**Published:** 2022-12-30

**Authors:** Michael Oertel, Dominik Hering, Nina Nacke, Christopher Kittel, Kai Kröger, Jan Kriz, Michael Fuchs, Christian Baues, Dirk Vordermark, Rita Engenhart-Cabillic, Klaus Herfarth, Peter Lukas, Heinz Schmidberger, Simone Marnitz, Peter Borchmann, Andreas Engert, Uwe Haverkamp, Hans Theodor Eich

**Affiliations:** a Department of Radiation Oncology, University Hospital Muenster, Muenster, Germany; b Department of Radiation Oncology, Alexianer Clemenshospital Muenster, Muenster, Germany; c Department of Internal Medicine, Center for Integrated Oncology Aachen Bonn Cologne, Düsseldorf, University Hospital of Cologne, Cologne, Germany; d Department of Radiation Oncology and Cyberknife Center, University Hospital of Cologne, Cologne, Germany; e Department of Radiation Oncology, University Hospital Halle (Saale), Halle (Saale), Germany; f Department of Radiotherapy and Radiation Oncology, University Hospital Giessen-Marburg, Marburg, Germany; g Department of Radiation Oncology, Heidelberg University Hospital, Heidelberg, Germany; h Department of Radiooncology, Medical University Innsbruck, Innsbruck, Austria; i Department of Radiotherapy and Radiation Oncology, University Hospital Mainz, Mainz, Germany

## Abstract

**Purpose:**

Radiation therapy (RT) is an integral part of treatment concepts for early-stage Hodgkin lymphoma. This analysis reports on RT quality in the recent HD16 and 17 trials of the German Hodgkin Study Group (GHSG).

**Methods and Materials:**

All RT plans of involved-node radiation therapy (INRT) in HD 17 were requested for analysis, along with 100 and 50 involved-field radiation therapy (IFRT) plans in HD 16 and 17, respectively. A structured assessment regarding field design and protocol adherence was performed by the reference radiation oncology panel of the GHSG.

**Results:**

Overall, 100 (HD 16) and 176 (HD 17) patients were eligible for analysis. In HD 16, 84% of RT series were evaluated as correct, with significant improvement compared with the predecessor studies (*P* < .001). In HD 17, 76.1% of INRT cases revealed a correct RT design compared with 69.0% of IFRT-cases, which was superior to previous studies (*P* < .001). Comparing INRT and IFRT, we found no significant differences in the percentage of any deviation (*P* = .418) or major deviations (*P* = .466). Regarding dosimetry, INRT was accompanied by an improvement in thyroid doses. Comparing different RT techniques, we found that intensity-modulated RT showed a reduction of high doses in the lung at the expense of an increased low-dose exposure in HD 17.

**Conclusions:**

The latest study generation of the GHSG demonstrates an improved quality in RT. A modern INRT design could be established without deterioration in quality. On a conceptual level, an individual consideration of the appropriate RT technique has to be performed.

## Introduction

Radiation therapy (RT) has been an integral part of different treatment schedules for Hodgkin lymphoma (HL) since the introduction of large-field techniques in the second half of the 20th century.^1^ Consequently, the technical evolution of RT, the implementation of systemic chemo- and immunotherapy, and the deepening of understanding concerning the disease's biology has led to a continuous decrease both in RT field size and dose.^2^^,^^3^ Today, a combined modality approach is applied using multiagent chemotherapy regimens like ABVD (doxorubicin, bleomycin, vinblastine, dacarbazine) or BEACOPP (bleomycin, etoposide, doxorubicin, cyclophosphamide, vincristine, procarbazine and prednisone), followed by consolidative RT.^4^^,^^5^ Involved-node radiation therapy (INRT) or involved-site radiation therapy (ISRT) is considered state of the art for first-line treatment with a dose of 20 to 30 Gy adapted to disease stage and risk category.^4^, ^5^, ^6^ Recent randomized trials questioned the role of RT by introducing chemotherapy-only treatment concepts to early-favorable and early-unfavorable/intermediate-stage HL, respectively, but failed to demonstrate therapeutic equivalency of the monomodal treatment.^7^^,^^8^ The HD 16 and HD 17 trials represent the latest generation of phase 3 trials launched by the German Hodgkin Study Group (GHSG) addressing early- or intermediate-stage HL, respectively.^9^^,^^10^ In the HD 16 trial, patients with HL in an early-favorable stage were randomized after 2 cycles of ABVD chemotherapy between a standard arm of 20 Gy of involved-field radiation therapy (IFRT) and a positron emission tomography (PET)–adapted arm, in which RT was limited to PET-positive cases^9^ The HD 17 trial randomized patients with HL in intermediate/early-unfavorable stage after upfront chemotherapy of 2 cycles of escalated BEACOPP and 2 cycles of ABVD between a standard treatment (30 Gy IFRT) and an experimental approach.^10^ The latter stratified patients according to PET status, with an additional 30 Gy INRT in cases of PET positivity and no further treatment for complete metabolic remissions. These modern treatment strategies account for the biology of HL on an individual level and direct RT to high-risk patients. However, the quality of RT planning and treatment setup in these trials have not been studied in detail. Previous analyses on quality assurance have illustrated the importance of a systematic assessment of RT plans leading to harmonization and improvement of treatment concepts.^11^, ^12^, ^13^, ^14^ The aim of the present analysis is to continue this work and to examine whether the high standards of the GHSG could be maintained. One essential question was whether the transfer from IFRT to INRT within HD 17 was successful. In addition, information on organs-at-risk delineation and dosimetric data are provided.

## Methods and Materials

### Study concepts

#### HD 16

The phase 3 international HD 16 study (NCT00736320; EudraCT code: 2007-004474-24) included more than 1100 patients with newly diagnosed HL aged between 18 and 75 years in Ann Arbor stage I or II without any risk factor (mediastinal bulk, extranodal involvement, elevated erythrocyte sedimentation rate, 3 or more involved nodal areas). Treatment consisted of 2 cycles of ABVD followed by a fluorodeoxyglucose–PET scan. In the standard arm, all patients received 20 Gy IFRT, whereas patients in the experimental arm had RT only in case of a PET-positive result (Deauville score ≥3). Results of the main trial showed a deterioration of progression-free survival (PFS; 86.1% vs 93.4% at 5 years) with the omission of RT.^9^

#### HD 17

The phase 3 HD 17 trial (NCT01356680; EudraCT code: 2007-005920-34) enrolled 1100 patients with early-stage unfavorable HL aged between 18 and 60 years in Ann Arbor stage I-IIA with any combination of the aforementioned risk factors. In addition, patients in Ann Arbor stage IIB with an elevated erythrocyte sedimentation rate and/or at least 3 nodal areas were included. Patients underwent 2 cycles of escalated BEACOPP and 2 cycles of ABVD and subsequent 30 Gy IFRT in the standard arm. In the experimental arm, RT was administered to patients with PET-positive results after chemotherapy as 30 Gy INRT. This PET-stratified approach proved to be non-inferior regarding PFS (5-year PFS: 97.3% [combined modality] vs 95.1% [PET guided]).^10^

### RT quality analysis

RT quality analysis was an integral part of the study protocol and covered by the respective institutional review board consents. The study goal was to analyze all patients with INRT treatment in HD 17. With IFRT being an established concept, only a randomized selection of patients undergoing this treatment were taken into analysis. In HD 16, 100 patients were analyzed.

### Panel evaluation

**Figure 1:**
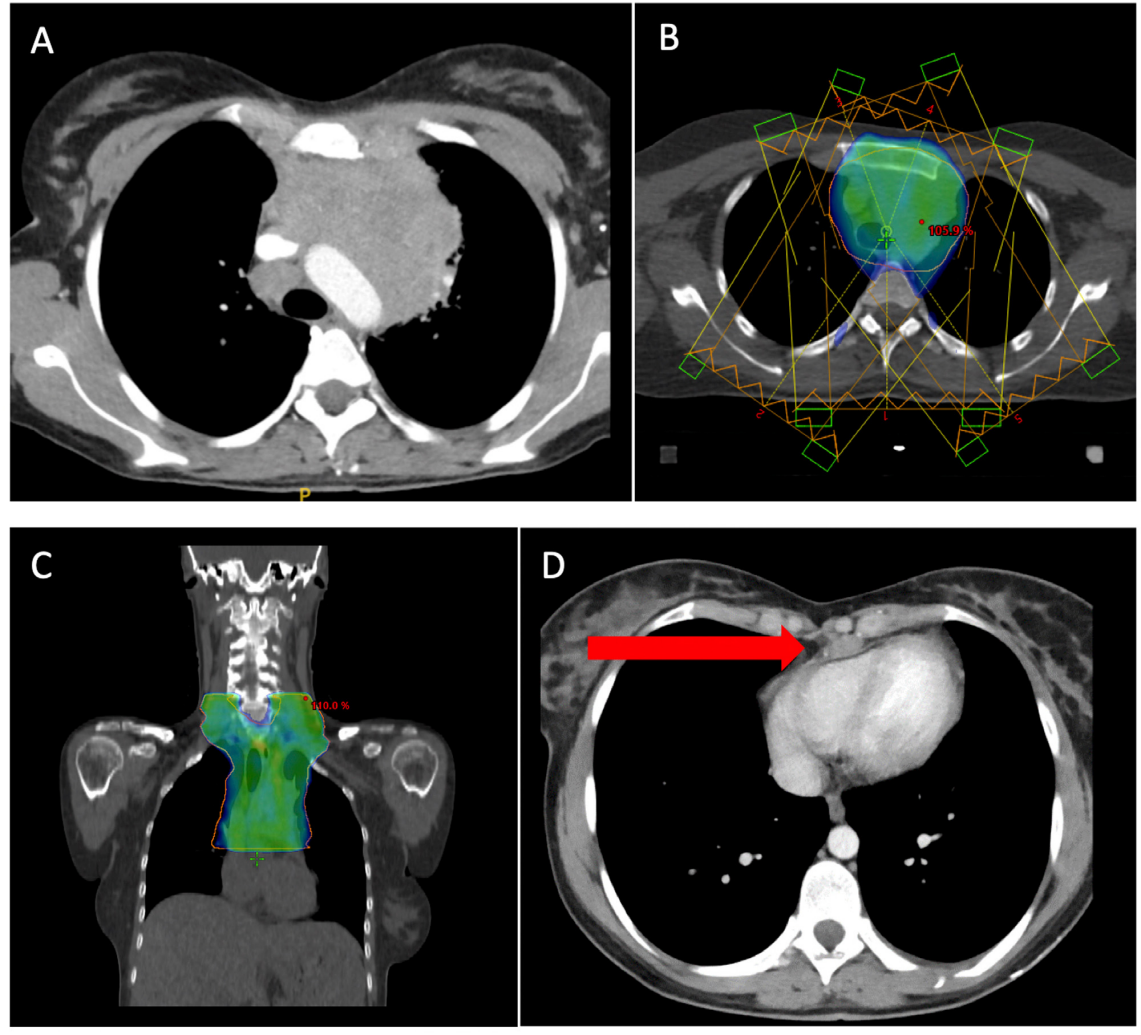
Example of a major deviation. (A) Prominent mediastinal bulk in a 30-year-old female patient showing displacement of arterial vessels. (B) Radiation treatment plan was executed as a 5-field intensity-modulated radiation therapy. Representation of the 95% isodose in color wash. (C) Coronal view of the mediastinal field. (D) A caudal precardial lymph node was not encompassed by the radiation field, constituting a major deviation.

All available RT series were assessed by the radiation oncology reference panel of the GHSG, the members of which are authors of the presented paper. To account for different opinions, at least 3 head of departments had to be present at each meeting. The evaluation process included the initial (pre-chemotherapy) imaging, RT plans, as well as recommendation from one of the reference radiation oncology institutions. During the structured process, target volume definition, RT techniques and setup, (fraction and total) doses, adherence to the reference RT recommendation, and correct execution of the plan were taken into account. Evaluation results were graded as “no deviation,” “minor deviation,” or “major deviation,” respectively, according to the study protocol. A “major deviation” describes relevant and severe violations of the study protocol that may endanger patient safety or treatment efficacy, for instance, too narrow field delineations in an involved region or dose deviations greater than 10% (Fig. 1). In comparison, “minor deviation” is used for any (negligible) difference from the study protocol not fulfilling the criteria of a major event. Evaluations were discussed in a multilevel process until a consensus was reached. First, panel rounds were held as meetings at Muenster, Germany. With the advent of the COVID-19 pandemic, a transfer to digital conferences had to be performed.

### Dosimetric analysis

Dose-volume histograms of the RT plans were analyzed for dosimetric information either as a paper-based evaluation or digitally. Subsequently, organs at risk (OAR) as well as dosimetric parameters (D_mean_ or D_max_, respectively) were registered and compared between patients receiving IFRT and INRT and different treatment techniques. For supradiaphragmatic RT fields, dose values for the spinal cord, left and right lung, esophagus, heart, parotid glands, thyroid glands, female breast, and coronary vessels were evaluated, whereas the analysis for infradiaphragmatic RT field encompassed the spinal cord, kidneys, bowel, and gonads.

### Statistical analysis

Continuous variables are summarized by the minimum, median, and maximum values, compared with categorical variables being presented as absolute numbers or relative frequencies. Normal distributions were assessed using a Shapiro–Wilk test. According to the presence of normal distribution, a 2-sample *t-*test or a Mann–Whitney *U* test was used to compare mean or median values or ranks, respectively. For these analyses, exact significances were considered. Distributions were compared using a Kolmogorov–Smirnov test. A χ^2^ test was used for comparisons between different evaluation results testing for 2-sided exact significance. In all cases, a *P* value < .05 was considered to be statistically significant. All statistical analyses were carried out using Microsoft Excel (Microsoft, Redmond, WA) and SPSS version 28 (IBM, Armonk, NY).

## Results

### Panel evaluation

#### HD 16

Overall, 100 patients undergoing IFRT were analyzed. Radiation doses were adequate with a median of 20 Gy (19.8-21.6 Gy) in normofractionation. In the majority of radiation series, supradiaphragmatic target volumes were treated (91%), with the regions most commonly irradiated being supraclavicular left/right (73% each), infraclavicular left/right (73% each), and cervical left/right (44% and 45%, respectively). According to the RT panel, 84% of cases were evaluated as “correct,” 5% as “minor deviations,” and 11% as “major deviations.” Major deviations were caused by insufficient dose coverage of involved regions (11/11), predominantly in the upper mediastinum (5/11 cases; Table 1). Previous GHSG studies in early-stage HL (HD 10^12^ and HD 13^11^) showed a lower number of RT series executed according to protocol (38.8% and 52%, respectively). A χ^2^ test was used to compare the number of correct RT series in the different GHSG study generations (HD 13 vs HD 16). No expected cell frequencies were less than 5. Results reveal a significant improvement in favor of HD 16 (χ^2^[1] = 33.807, *P* < .001, φ = –0.247). A similar improvement was found concerning the absence of major deviations in relation to the GHSG study generation (χ^2^[1] = 27.378, *P* < .001, φ= –0.222).

**Table 1:** Panel evaluations of HD 16/17

Evaluation	HD 16	HD 17 (IFRT)	HD 17 (INRT)
Correct/no deviation	84% (84/100)	69.0% (29/42)	76.1% (102/134)
**Major deviations**	11% (11/100)	19.0% (8/42)	14.2% (19/134)
Insufficient coverage of an involved region	11% (11/100)	14.3% (6/42)	12.7% (17/134)
Incorrect RT dose	0% (0/100)	2.4% (1/42)	1.5% (2/134)
Confusion of sides	0% (0/100)	2.4% (1/42)	0% (0/134)
**Minor deviations**	5% (5/100)	11.9% (5/42)	9.7% (13/134)*
Insufficient coverage of an adjuvant region	5% (5/100)	4.8% (2/42)	2.2% (3/134)
PTV too large	0% (0/100)	4.8% (2/42)	4.5% (6/134)
Incorrect PTV definition (margins, adaptions)	0% (0/100)	0% (0/42)	1.5% (2/134)
Incorrect RT setup	0% (0/100)	2.4% (1/42)	0% (0/134)
Excessive beam energy	0% (0/100)	0% (0/42)	0.7% (1/134)
Insufficient RT (fraction) dose	0% (0/100)	0% (0/42)	1.5% (2/134)

*Abbreviations:* IFRT = involved-field radiation therapy; INRT = involved-node radiation therapy; PTV = planning target volume; RT = radiation therapy.

RT plans were evaluated as “correct/no deviation,” “minor deviation,” or “major deviation,” respectively (see text for further details). Percentages of the respective categories with absolute numbers given in parentheses. *In the INRT arm of HD17, there was one case with excessive beam energy and a too large PTV, thus subcategories do not add up to 9.7%.

#### HD 17

In total, 176 patients (INRT: 134, IFRT: 42) were analyzed and treated with a median RT dose of 30 Gy (IFRT: 18-30.6 Gy, INRT: 14-40 Gy). Overall, 76.1% of INRT cases showed no deviation compared with 69.0% of IFRT cases. Deviations were reported for 9.7% and 14.2% of patients in the INRT group compared with 11.9% and 19.0% in the IFRT group for minor and major deviations, respectively. There was no significant difference between both cohorts regarding the percentage of plans with deviations (*P* = .418) or the percentage of major deviations (*P* = .466). The principal causes for major deviations were too narrow target volumes in the involved region (IFRT: 6 vs INRT: 17) or incorrect RT doses (IFRT: 1, INRT: 2; Table 1). In comparison with the HD 11 and HD 14 trial, there was a continuous increase in the percentages of RT series performed according to protocol (33.0% in HD 11 vs 37.8% in HD 14 vs 74.4% in HD 17 [pooled data for INRT and IFRT]). Again, a χ^2^ test was applied for comparisons between the different GHSG study generations (HD 14 vs HD 17). No expected cell frequencies were less than 5. The results underlined a significant improvement in favor of the recent study generation (χ^2^[1] = 71.045, *P* < .001, φ = –0.310). Similar outcomes were found for the association of GHSG study generation and the absence of major deviations (χ^2^[1] = 75.232, *P* < .001. φ = –0.319).

### Comparison between INRT and IFRT

The study design of HD17 enabled a dosimetric comparison between INRT and IFRT (Table 2). For most parameters, no significant advantage of INRT could be found. For planning target volume (PTV), the spinal cord and V_25_ of the right lung, a statistically significant difference in dose or volume exposure between the 2 groups could be shown, respectively (spinal cord: U = 947.5, Z = –3.527, *P* < .001; PTV: U = 1367.0, Z = –32.021, *P* = .043; V_25_ of the right lung: U = 939.0, Z = –32.099, *P* = .035). The distributions for these parameters differed between both groups (Kolmogorov–Smirnov, *P* < .05). Furthermore, there have been significant dosimetric advantages in favor of INRT for V_25_ of the left lung (*P* = .024) as well as D_mean_ and V_25_ of the thyroid (*P* = .023 and 0.048, respectively).

**Table 2:** Dosimetric analysis for supradiaphragmatic organs at risk in HD17 according to the field design

	Total cohort	IFRT (HD 17)	INRT (HD 17)	*P*
No.	146	35	111	
PTV, mL	1265.2 (86.7-5125.3)	1464.3 (97.6-4238.0)	1163.1 (86.7-5125.3)	**.043**
Lung right D_mean_	9.8 (0.3-20.0)	9.4 (4.0-17.8)	10.1 (0.3-20.0)	.832
V_5_ (%)	55.0 (0-100)	48.5 (15.9-98.0)	57.1 (0-100)	.319
V_10_ (%)	38.0 (0.0-86.0)	35.0 (12.1-86.0)	38.0 (0-82.5)	.534
V_20_ (%)	20.9 (0.0-50.0)	21.3 (6.0-46.0)	19.7 (0-50.0)	.150
V_25_ (%)	13.3 (0.0-42.0)	16.4 (3.0-40.0)	12.3 (0-42.0)	**.035**
V_30_ (%)	2.0 (0.0-31.9)	2.9 (0-30.0)	1.5 (0-31.9)	.095
Lung left D_mean_	10.5 (0.2-26.5)	11.3 (1.0-23.5)	10.1 (0.2-26.5)	.290
V_5_ (%)	58.0 (0.0-99.0)	50.4 (1.5-98.5)	59.2 (0-99.0)	.620
V_10_ (%)	38.8 (0.0-92.0)	39.8 (0.0-90.0)	37.8 (0.0-92.0)	.942
V_20_ (%)	20.9 (0.0-85.0)	23.4 (0.0-70.3)	19.7 (0.0-85.0)	.105
V_25_ (%)	14.2 (0.0-80.0)	16.9 (0.0-62.0)	12.5 (0.0-80.0)	**.024**
V_30_ (%)	2.0 (0.0-60.0)	4.2 (0.0-30.0)	2.0 (0.0-60.0)	.086
Spinal cord D_max_	29.6 (6.9-34.2)	31.2 (15.6-34.2)	28.7 (6.9-32.6)	**<.001**
Esophagus D_mean_	21.4 (8.9-30.0)	24.9 (10.1-30.0)	20.5 (8.9-29.5)	.119
Heart D_mean_	13.1 (0.5-30.4)	14.4 (0.6-30.4)	12.4 (0.5-26.9)	.691
Thyroid D_mean_	26.5 (13.9-33.3)	31.1 (29.4-33.3)	24.2 (13.9-31.3)	**.023**
Thyroid V_25_ (%)	55.0 (35.0-100)	100 (98.5-100)	43.8 (35-100)	**.048**
Breast left D_mean_	3.7 (0.4-15.6)	3.9 (2.1-10.9)	3.7 (0.4-15.6)	.476
Breast right D_mean_	3.6 (0.5-9.3)	4.3 (1.0-6.8)	3.5 (0.5-9.3)	.935

*Abbreviations:* IFRT = involved-field radiation therapy; INRT = involved-node radiation therapy; PTV = planning target volume.

Median values in Gray are given with the minimum and maximum values in parentheses afterwards. There was 1 case with 2 PTV in both treatment groups. *P* values are given for the Mann–Whitney *U* and 2-sample *t-*test, respectively, according to the presence of a normal distribution. Significant values are shown in bold.

### RT technique

In HD 16, RT was executed as 3-dimensional conformal RT (3D-CRT) in 76 patients, intensity-modulated RT (IMRT; including volumetric modulated arc therapy and tomotherapy) in 18 patients, or a combination of modalities (2, with 4 being unknown). In HD 17, numbers were 28 and 67 for 3D-CRT compared with 13 and 66 cases of IMRT treatment in the IFRT and INRT group, respectively. One case in the IFRT arm had an unknown treatment and 1 case in the INRT underwent a combined treatment.

Comparing the conventional RT techniques with modern IMRT revealed different dose exposures. For patients in HD 16, lung volumes exposed to 20 Gy were reduced with IMRT (median right: 1.6 vs 0.0 Gy, *P* = .014; median left: 3.3 vs 0.0 Gy, *P* = .006; Table 3). For the spinal cord, distribution of values differed between the 2 groups (*P* < .05 in the Kolmogorov–Smirnov test), and significant difference could be found (difference 3D-CRT: mean rank 39.33 vs IMRT: 15.7, *P* < .01, U = 102.0, Z = –3.356). In addition, technical comparisons between 3D-CRT and IMRT in HD 17 elaborated a significant increase in V_5_ and V_10_ but concomitant decrease of V_20_, V_25_ and V_30_ with the use of IMRT (Table 4).

**Table 3:** Dosimetric data for IFRT in HD 16 and subgroup analysis of patients treated with 3D-CRT and IMRT, respectively

	HD16 (IFRT)	3D-CRT	IMRT	*P*
No.	100	76	18	
PTV, mL	956.1 (198.2-3253.3)	1031.7 (280.0-3253.3)	755.5 (198.2-1500.4)	.088
Lung right D_mean_	4.3 (0.2-9.2)	4.4 (0.2-9.2)	4.3 (1.0-6.5)	.389
V_5_ (%)	22.1 (0.0-50.0)	22.1 (0-50.0)	19.7 (0.0-45.0)	.952
V_10_ (%)	17.7 (0.0-43.0)	17.7 (0.0-43.0)	13.4 (0.0-23.0)	.300
V_20_ (%)	1.0 (0.0-19.0)	1.6 (0.0-19.0)	0.0 (0.0-2.0)	**.014**
Lung left D_mean_	4.7 (0.2-15.2)	5.3 (0.2-15.2)	3.9 (0.2-7.0)	.187
V_5_ (%)	27.5 (0.0-84.0)	30.5 (0.0-84.0)	26.3 (0.0-52.5)	.565
V_10_ (%)	20.0 (0.0-79.0)	22.4 (0.0-79.0)	10.0 (0.0-33.0)	.164
V_20_ (%)	1.8 (0.0-20.0)	3.3 (0.0-20.0)	0.0 (0.0-3.3)	**.006**
Spinal cord D_max_	20.5 (2.3-22.3)	20.7 (2.3-22.3)	15.2 (9.1-20.0)	**<.001**
Esophagus D_mean_	13.9 (3.4-21.0)	13.6 (3.4-20.1)	18.2 (6.4-21.0)	.427
Heart D_mean_	3.8 (0.0-16.0)	4.2 (0.0-16.0)	3.1 (1.1-5.7)	.812
Thyroid D_mean_	19.6 (2.1-20.2)	19.6 (2.1-20.2)	-	-
Breast left D_mean_	1.0 (0.3-4.0)	0.3 (0.3-4.0)	1.7 (1.7-1.7)	-
Breast right D_mean_	0.3 (0.2-4.7)	0.25 (0.2-0.3)	4.7 (4.7-4.7)	-

*Abbreviations:* 3D-CRT = 3-dimensional conformal radiation therapy; IFRT = involved-field radiation therapy; IMRT = intensity-modulated radiation therapy; PTV = planning target volume.

Median values in Gray are given with the minimum and maximum values in parentheses afterwards. *P* values are given for the Mann-Whitney *U* and 2-sample *t-*test, respectively, according to the presence of a normal distribution. Significant values are shown in bold. Patient numbers were too small to analyze values for left and right breast, respectively as well as the thyroid gland.

**Table 4:** Dosimetric data in HD 17 according to RT treatment technique

	3D-CRT	IMRT	*P*
No.	95	79	
PTV, mL	1297.2 (297.1-4238.0)	1292.5 (252.6-5125.3)	.838
Lung right D_mean_	9.1 (2.4-20.0)	10.4 (0.3-16.6)	.365
V_5_ (%)	45 (12.4-88.1)	66.4 (0.0-100.0)	**<.001**
V_10_ (%)	32.0 (0.0-72.0)	44.8 (0.0-86.0)	**.006**
V_20_ (%)	22.0 (0.0-50.0)	17.5 (0.0-35.1)	**<.001**
V_25_ (%)	18.0 (0.0-42.0)	8.1 (0.0-23.2)	**<.001**
V_30_ (%)	4.0 (0.0-31.9)	1.0 (0.0-12.0)	**<.001**
Lung left D_mean_	9.6 (0.2-26.5)	11.0 (2.0-20.8)	.098
V_5_ (%)	45 (0.0-97.0)	64.8 (11.0-99.0)	**<.001**
V_10_ (%)	34.2 (0.0-92.0)	45.5 (0.0-90.0)	**.001**
V_20_ (%)	23.3 (0.0-85.0)	18.9 (0.0-51.8)	**.041**
V_25_ (%)	18.9 (0.0-80.0)	10.7 (0.0-36.9)	**<.001**
V_30_ (%)	3.2 (0.0-60.0)	1.1 (0.0-19.0)	**<.001**
Spinal cord D_max_	31.2 (6.9-34.2)	25.4 (14.0-32.9)	**<.001**
Esophagus D_mean_	23.4 (10.1-30.0)	20.1 (8.9-29.3)	**.041**
Heart D_mean_	13.6 (0.6-30.4)	11.2 (0.5-22.8)	.258
Thyroid D_mean_	30.8 (13.9-33.3)	25.7 (20.1-31.1)	.535
Thyroid V_25_ (%)	70.0 (35.0-100.0)	55.0 (42.0-100.0)	.930
Breast left D_mean_	3.4 (0.4-7.9)	4.7 (1.2-15.6)	.089
Breast right D_mean_	2.7 (0.5-9.3)	4.0 (0.5-8.9)	.314

*Abbreviations:* 3D-CRT = 3-dimensional conformal radiation therapy; IMRT = intensity-modulated radiation therapy; PTV = planning target volume; RT = radiation therapy.

Comparison between 3D-CRT and IMRT, respectively. Median values in Gray are given with the minimum and maximum values in parentheses afterward. *P* values are given for the Mann–Whitney *U* and 2-sample *t*-test, respectively, according to the presence of a normal distribution. Significant values are shown in bold.

#### Organs at risk delineation

Information on supradiaphragmatic OAR could be retrieved for 79 patients in HD 16 and 146 patients in HD 17 and revealed heterogeneity in contouring (Table 5). Rates of contouring greater than 90% were found for the spinal cord, whereas numbers dropped considerably for breast tissue (3.8%-21.6% for left and right breast in HD16 and the 2 arms of HD17, respectively). There was no case in which the coronary vessels were contoured.

**Table 5:** Contouring of organs at risk

Organ	INRT (HD 17)	IFRT (HD 17)	IFRT (HD 16)
Spinal cord	97.3% (109/111)	91.4% (32/35)	94.9% (75/79)
Lung left	100% (112/111)	91.4% (32/35)	73.4% (58/79)
Lung right	100% (112/111)	91.4% (32/35)	78.5% (62/79)
Esophagus	32.4% (36/111)	22.9% (8/35)	17.7% (14/79)
Heart	92.8% (103/111)	65.7% (23/35)	34.2% (27/79)
Parotid gland left	28.8% (32/111)	57.1% (20/35)	29.1% (23/79)
Parotid gland right	28.8% (32/111)	60% (21/35)	29.1% (23/79)
Thyroid	9.0% (10/111)	11.4% (4/35)	7.6% (6/79)
Breast left	20.7% (23/111)	20% (7/35)	5.1% (4/79)
Breast right	21.6% (24/111)	20% (7/35)	3.8% (3/79)
Coronary vessels	0% (0/111)	0% (0/35)	0% (0/79)

*Abbreviations:* IFRT = involved-field radiation therapy; INRT = involved-node radiation therapy.

Percentages and absolute numbers (in parentheses) for the contouring and dose availability of the respective organ.

With the majority of patients undergoing supradiaphragmatic treatments, only sparse information on infradiaphragmatic dose exposure were available. Overall, there were 11 patients in HD 17 (3 IFRT and 8 INRT) and 9 patients in HD 16 with involvement below the diaphragm. Most commonly, the spinal cord and both kidneys were contoured (100% in HD 17, but only 11.1% or 22.2% in HD 16), but only a minority of cases (HD 16: 0%, HD 17: 20%) revealed bowel or gonads.

### Toxicity

In both studies, acute toxicities during RT were mild to moderate, with only 3 cases of grade 3 toxicities in HD 16 (1 nausea or vomiting, 1 dysphagia, 1 mucositis). In HD 17, 3 cases of dysphagia occurred in the INRT and IFRT arms, respectively. One of these patients in the IFRT cohort had additional grade 3 mucositis and one additional patient in the INRT arm suffered from grade 3 to 4 leukopenia.

## Discussion

The hereby presented analysis demonstrates a high quality of RT for HL in the modern era. It is the first to document and analyze the paradigm shift from IFRT to INRT revealing constancy in quality. Furthermore, it illustrates the challenges to comply with restrictive dose constraints in the modern era.

In the past decades, the use of RT in stage I and II has declined significantly according to an analysis from the Surveillance, Epidemiology, and End Results database (62.9% in 1988-1991 vs 43.7% in 2004-2006; *P* < .001).^15^ The British Rapid Trial, the European H10 trial and the GHSG HD 16 trials attempted to omit RT in the treatment schedule but could not demonstrate noninferiority of the chemotherapy-only regimens.^7^, ^8^, ^9^ The experimental arms of these trials were driven by the idea to maintain therapeutic equivalency but to reduce long-term toxicity, as secondary malignancies and cardiovascular diseases are the predominant mortality risks in the second decade after lymphoma treatment.^16^^,^^17^ Although both infra- and supradiaphragmatic RT are described as risk factors for secondary cancers, no decrease in their incidence could be observed in the modern era.^17^, ^18^, ^19^ In contrast, at least one study points toward a decrease in 25-year cardiovascular treatment mortality in the recent treatment period (4.3% for patients treated during 1989-2000 vs 5.7% for treatment in 1965-1976).^19^ Data from modern ISRT and INRT concepts are still lacking. Moreover, most studies outline no decisive analysis of RT field designs and rather present a dichotomous stratification, analyzing the presence or absence of RT.

The HD 17 trial is the only randomized trial in which both an IFRT and an INRT concept are present in the study arms. Information on accurate target volume delineation was provided in the study protocol and specified in accompanying publications.^20^ Surprisingly, the introduction of the modern and smaller INRT treatment did not result in a significant dosimetric reduction for most OAR (Table 2). One reason for this may be the rather conservative clinical target volume to PTV margin used in HD 17, with a 2-cm margin in axial dimension and 3 cm for cranial–caudal expansion.^20^ In contrast, the European Organisation for Research and Treatment of Cancer used a 1-cm margin for their definition of INRT^21^ and outlined a successful reduction of recurrences in previously involved areas in their H10 trial.^7^ In the favorable and unfavorable arm of this study, only none and 5 recurrences in an initially involved lymph node area occurred after a combined treatment of chemo- and radiotherapy, respectively.^7^

Regarding RT techniques, our analysis demonstrates the advantage of IMRT in avoiding high doses in organs like the lung, spinal cord, or thyroid at the expense of a greater low-dose exposure (Table 3 and 4). This finding has been reported previously and may contribute to a greater risk of secondary malignancies in the long-term follow-up.^22^^,^^23^ Therefore, a careful and individual risk–benefit analysis has to be performed without general recommendation of a uniform RT strategy as reflected by modern guidelines.^24^

Historically, quality assurance by the reference radiology and radiation oncology panel of the GHSG has proven to enhance both accuracy in diagnostic and therapy for HL.^11^^,^^12^^,^^14^ The main reason for major deviations still lie within an inadequate coverage of an involved region which is in accordance with the literature.^11^^,^^12^ A detailed recurrence analysis of the HD 16 trial showed that infield relapses constitute the major pattern of failures if RT is omitted in the treatment.^25^ On the contrary, adequate IFRT was able to reduce infield failures from 8.7% to 2.1%.^25^ Importantly, inaccurate target volume coverage in HL has been identified as a risk factor for relapse. In an analysis by Kinzie et al,^26^ incorrect field margins resulted in a recurrence rate of 50% in comparison with 15% in case of a correct setup.^26^ Likewise, relevant protocol violations of RT in the GHSG HD 4 trial led to a decline in relapse-free survival (72% vs 84% at 7 years, *P* = .0043).^13^ However, in comparison with the predecessor studies, the major deviation rates in RT planning in HD 16/17 were lower (11% and 16.7% for HD 16/17 vs 36.8% and 42.5% for HD 10/11 vs 36% and 45% for HD 13/14), illustrating a learning curve for the delineation of IFRT.^11^^,^^12^ This development was enabled by a continuous educational effort conducted by our group, which includes workshops but also contouring sessions and refresher courses at the annual meeting of our national radiation oncology society. In this regard, the introduction of INRT was not accompanied by a deterioration in quality, as no significant differences to the IFRT arm could be found (*P* = .418 and *P* = .466 for overall and major deviations, respectively). Further protocol violations due to technical reasons, incorrect setup, or (fraction) dose were less prevalent in both arms. Surprisingly, there was a reduced rate of correct IFRT series in HD 17 compared with HD 16, the reasons of which may be only speculated upon. It is possible the study centers struggled to define adequate INRT fields being neither too large nor too narrow in a direct comparison with the more precise INRT.

Concerning OAR delineation, there was a considerable heterogeneity in contouring between the different organs with varying percentages. Although some OAR may have not been required in every case, for example, the parotid glands in case of a mediastinal involvement, some inconsistency remains, for instance, a contouring of both lungs while lacking the heart or female breasts. Particularly, there was no case of coronary artery contouring. For this OAR, a linear dose-side effect relationship has been described with a 7.4% increase of coronary heart disease for every additional Gy of mean heart dose .^27^ Therefore, dose maxima in the coronary arteries have to be avoided, keeping the dose as low as reasonably possible.^28^ Inadequate contouring may bias the dosimetric results presented here: only contoured organs can be accounted for in the RT planning process, and this may blur potential differences, for instance, in the comparison between IMRT and 3D-CRT. Therefore, systematic and detailed contouring should be a major focus for further educational activities.

Dose constraints have been reported by the International Lymphoma Radiation Oncology Group.^28^ Ideal doses include a mean heart dose <5 Gy, a mean breast dose <4 Gy, a V_5_ <55%, and V_20_ <30% in the lung, a mean lung dose <10 Gy, as well as a V_25_ of the thyroid <62.5%. When using 30 Gy in HD 17, some constraints were not met, for example, V_5_ in the lungs or the mean heart dose, underlining the importance of a careful RT planning.

As a consequence of the low RT doses used in the protocols, grade 3 toxicities were rare, with only 11 events in both studies. Correspondingly, grade 3 or 4 toxicities were registered in a total of 3% to 26% of cases in the study arms of HD 16 and HD 17, respectively.^9^^,^^10^ Focusing on RT toxicity, only 3.4% of patients in HD 16 suffered from grade 3 side effects, the majority being dysphagia (1.8%) and mucositis (0.9%).^25^

The presented study reveals some limitations. Despite all efforts, it was not possible to obtain all INRT plans from HD 17 due to an incomplete response rate. Furthermore, the number of patients with infradiaphragmatic disease was limited, which prevented a decisive sub-analysis. The number of IFRT cases analyzed was intentionally limited, which may lead to false assessment of failure rates. However, as the respective percentages are both in line with HD 16 and the INRT cohort of HD 17, this suggestion is unlikely. In addition, a matched cohort analysis between INRT and IFRT could not be established due to limited patient numbers. Technically, the HD 16/17 trials were conducted during a period in which IMRT was not used routinely in many departments. Thus, advanced techniques like butterfly volumetric arc therapy were only applied in rare cases.^29^ Future workshops may help to spread the knowledge on these techniques and enable a widespread application.

The results of HD 17 and the H10U by the European Organisation for Research and Treatment of Cancer established a risk-adapted treatment strategy for patients with intermediate-stage HL,^7^^,^^10^ which demands an individualized and modern RT conceptualization. This evolution will continue: In the recent GHSG NIVAHL study, immunotherapy was introduced in the first-line treatment, which will alter treatment responses and probably RT design.^30^ In the end, further long-term analyses will be needed to examine a possible correlation between RT quality and oncological outcomes in the context of INRT and ISRT. In the meantime, continuous educational activities are needed to maintain the high-quality of RT planning and execution demonstrated in this analysis.
